# CITN-06: a Phase I/expansion trial of alt-803, an IL-15 superagonist, in patients with advanced melanoma

**DOI:** 10.1186/2051-1426-2-S3-P79

**Published:** 2014-11-06

**Authors:** Kim Margolin, Hing C Wong, Chihiro Morishima, Liza Hernandez, Marc Ernstoff, Peter R Rhode, Thomas A Waldmann

**Affiliations:** 1 University of Washington / Stanford University, Stanford, CA, USA; 2 Altor BioScience Corporation, Miramar, FL, USA; 3 University of Washington, Seattle, WA, USA; 4 Dartmouth University, Hanover, NH, USA; 5 Lymphoid Malignancies Branch/CCR/NCI, Bethesda, MD, USA

## Background

IL-15 activates and induces the proliferation of CD8+ T cells and NK cells. ALT-803 (Altor Bioscience, Miramar, FL) is a superagonist of IL-15 consisting of 2 molecules of IL-15 N-to-D substituted at position 72, bound to 2 molecules of the "sushi" domain of IL-15 alpha receptor and a single Fc fragment of IgG1. ALT-803 has been shown to exert superior antitumor activity and possess a longer half-life compared with unmodified IL-15 in animal models of myeloma, melanoma and bladder cancer. The Cancer Immunotherapy Trials Network (CITN) has initiated a Phase I/expansion trial of weekly intravenous (i.v.) ALT-803 in patients (pts) with advanced melanoma (mel). The primary objectives are to identify a maximum tolerated dose (MTD)/minimum effective dose (MED), defined by toxicities and by lymphocyte and leukocyte expansion, respectively. A cohort of 12 pts will then receive Rx at the MTD or MED (whichever is lower) to further analyze immune responses and screen for antitumor activity. The overall goal is to develop a regimen that can be combined with immune checkpoint blockade, co-stimulatory molecules, adoptive T cell therapy, tumor vaccine or intralesional Rx.

## Methods

Dose escalation will be conducted by treating 1 pt each at the first 2 dose levels (0.3 and 0.5 mcg/kg weekly) followed by 3 or 6 pts at each of the higher dose levels (1, 3, 6 mcg/kg) until MTD or MED is reached. Each cycle consists of 4 weekly i.v. bolus infusions followed by 2 weeks' observation. Pts receive the first dose as inpatients for close hemodynamic monitoring and subsequent Rx in the outpatient setting. The absolute lymphocyte count and other laboratory and clinical parameters are evaluated weekly, and flow cytometric enumeration and functional analyses of T and NK cell subsets are done pre-treatment (Rx) and weekly.

## Results

To date, 2 pts have initiated Rx. The first pt, unmedicated for the first dose, experienced fever and rigor but no hemodynamic effects after the first dose and no further side effects after doses 2-4. The second pt, premedicated with acetaminophen, received the first dose with no side effects. Data regarding the peripheral blood T and NK cell responses to Rx will be presented. Pt accrual to dose level 3 will begin in August, 2014.

This study is supported by NIH 1U01 CA154967-01 (http://clinicaltrials.gov/ NCT01727076) and generous grants from Altor Bioscience and the Melanoma Research Alliance.

